# Deeply learned broadband encoding stochastic hyperspectral imaging

**DOI:** 10.1038/s41377-021-00545-2

**Published:** 2021-05-25

**Authors:** Wenyi Zhang, Hongya Song, Xin He, Longqian Huang, Xiyue Zhang, Junyan Zheng, Weidong Shen, Xiang Hao, Xu Liu

**Affiliations:** 1 State Key Laboratory of Modern Optical Instrumentation, College of Optical Science and Technology, Zhejiang University, Hangzhou, 310027 China; 2 Intelligent Optics & Photonics Research Center, Jiaxing Institute of Zhejiang University, Jiaxing, 314000 Zhejiang Province China

**Keywords:** Optical spectroscopy, Optoelectronic devices and components

## Abstract

Many applications requiring both spectral and spatial information at high resolution benefit from spectral imaging. Although different technical methods have been developed and commercially available, computational spectral cameras represent a compact, lightweight, and inexpensive solution. However, the tradeoff between spatial and spectral resolutions, dominated by the limited data volume and environmental noise, limits the potential of these cameras. In this study, we developed a deeply learned broadband encoding stochastic hyperspectral camera. In particular, using advanced artificial intelligence in filter design and spectrum reconstruction, we achieved 7000–11,000 times faster signal processing and ~10 times improvement regarding noise tolerance. These improvements enabled us to precisely and dynamically reconstruct the spectra of the entire field of view, previously unreachable with compact computational spectral cameras.

## Introduction

Spectral imaging provides a powerful sensing method for science, where spectral and spatial detection is simultaneously expected. Its applications include art conservation^1^, astronomy^2^, earth remote sensing^3^, biomedical engineering^4^, and atmospheric science^5^. However, traditional spectral imaging instruments have bulky mechanics for spectral dispersion^6^. Moreover, either spatial or spectral scanning is essential to generate a three-dimensional (3D) spectral-spatial data cube. These scanning processes dramatically extend the acquisition time. To overcome these limitations, the so-called “snapshot” computational spectral imaging technique was proposed^7–10^. In this regard, the basic idea was to place a coded mask in front of the dispersion prism^9^. However, this approach failed to reduce the volume because of its complex optical scheme. A more effective solution^10,11^ was to apply random spectral filters, which were initially developed for the spectrometer^12–14^. In contrast to classic optical filters with unique or multiple passing bands, random spectral filters refer to an array of filters featuring irregular and unrelated transitions/reflections^12^. Integrating these random spectral filters into cameras simultaneously with advanced computational algorithms allows the reconstruction of more spectral bands with fewer detection channels.

Nevertheless, the efficiency of the algorithm becomes the key driver of the snapshot system’s performance. The algorithms currently applied in these spectral cameras are generally extensions of the compressive sensing (CS) concept^15^. These methods require a sufficient number of random spectral filters to guarantee spectral resolution. For example, the hyperspectral cameras developed in previous studies^10,11^ usually have 25 or more filters to cover the entire visible region. Furthermore, these CS-based methods obtain sufficiently convergent results through an iterative process. Consequently, they are time-consuming, limiting the possible output data volume of the spectral camera. Therefore, the snapshot methods always have to sacrifice the spatial resolution to maintain a high reconstruction precision of the spectrum or vice versa. However, the derivation fails even under low-level noise. Extra efforts to digitally handle noise could extend the computing time and might induce artifacts if they are not appropriately employed.

Recently, deep learning methods have been adopted to improve reconstruction accuracy and speed^16,17^. However, their hardware still suffers from the large volume due to the coded mask and prism. For the compact random filter design, some deep learning approaches have been developed for both the target response definition^18,19^ and inverse design^20–23^. These methods work to some extent; however, none of them comprehensively considered both procedures. As such, their results are compromised either by the producibility of the designed spectral responses or by the sensitivity to the fabrication error^24^.

To address the problems of computational speed, noise tolerance, and filter design, we develop a new hyperspectral camera, namely broadband encoding stochastic (BEST) camera, based on a deep neural network (DNN)^25^ for both the filter design and the spectrum reconstruction. This study is organized as follows. First, we introduce the schematic of the BEST camera and address the filter design problem. Subsequently, we analyze the reconstruction speed and noise tolerance advantage of the proposed DNN. Then, we verify our proposal on two hyperspectral imaging architectures: passive mode (measuring the spectral radiance) and active mode (measuring the spectral reflectance). Finally, we discuss the reliability of our systems and summarize our work.

## Results

### Schematic of BEST camera

**Fig. 1:**
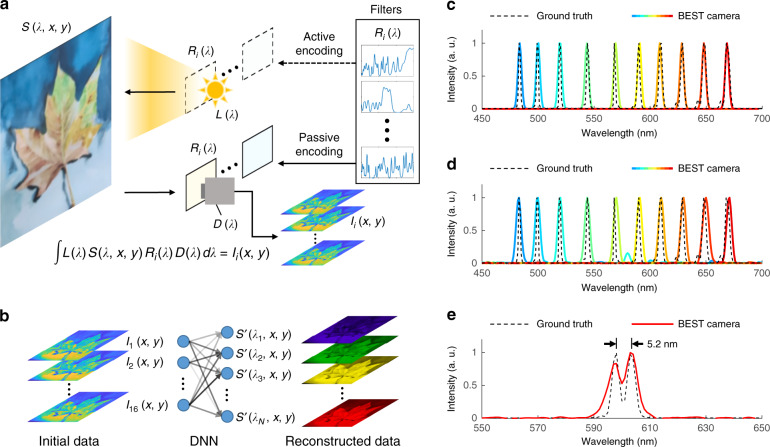
Principle and performance of BEST camera. **a** Simplified schematic. Depending on where the light spectrum is encoded, the camera can work either in the active (upper) or passive (lower) modes. **b** Principle of DNN-based spectral reconstruction algorithm. The initial data captured by the monochrome camera is fed into the DNN and outputs the reconstructed 3D hyperspectral data cube. **c**, **d** Spectral profiles of laser beams with narrow bandwidth. In **c**, the DNN is trained by the “precise” dataset, whereas **d** is for the results from the DNN trained by “general” datasets. **E** Spectral profile of two peaks corresponding to 598.0 nm and 603.2 nm. The peak-to-peak distance is highlighted in black. In **c**–**e**, the ground truths and the DNN reconstructed results are represented by dashed (ground truth) and solid (reconstructed) curves, respectively. The graphs are normalized to their peak intensity

A simplified schematic of the proposed device is shown in Fig. 1a. The proposed BEST camera is built using 16 random spectral filters. It has both active and passive modes. For the active mode, the filtered illumination spectrum encodes the sample; thus, the device detects the spectral reflectance of the sample $S(\lambda ,x,y)$. For the passive mode, the spectrum reflected by the sample is encoded by the filters; thus, the device detects the spectral radiance of the reflected light $L(\lambda )S(\lambda ,x,y)$. These filters were designed considering the parameter constrained spectral encoder and decoder (PCSED) method^24^ and fabricated by an electronic beam evaporator. In particular, as an extension of DNN for designing random spectral filters, PCSED focuses on obtaining the optimal spectral responsivity for each random spectral filter in the group, while ensuring their producibility. In brief, PCSED verifies large volumes of data to understand coating behaviors. Without manual intervention, it provides the coating design combinations maximizing the spectral resolution that are not apparent to humans. Using this strategy, we designed the proposed BEST camera with only 16 filters, corresponding to 40–60% fewer than those in previous studies^10,11^. The respective spectra and design details are available in Supplementary Information S1.

### Advantages of applying DNN

To extract the exact spectrum from these random spectral filters with rich features, we used the DNN for data processing (“Methods” section and Fig. 1b), which provides two major improvements over the original CS algorithms. First, the spectrum reconstruction speed can be orders of magnitude faster. This substantial acceleration is rooted, because the iterative algorithm solves an optimization problem for each pixel, whereas the DNN operates matrix multiplexing. The latter is especially suitable for parallel computing using a graphic processing unit (GPU). Therefore, for the iterative algorithm, the reconstruction time of a spectral image increases with increasing pixel number. However, the reconstruction speed of the DNN is almost pixel-independent. Although the speed is still comparable when the image only includes several thousand or fewer pixels, DNN is advantageous for images with higher resolution. To quantitatively compare the performance of the above two algorithms, we applied the proposed DNN to reconstruct a hyperspectral image (480 × 640 pixels, 1 nm spectral step size, 400–700 nm spectral scale) on an Intel Core i9-10900x CPU, Nvidia GeForce 2080Ti GPU platform. The DNN could generate such an image in 0.48 s. Although further optimization is possible, the current speed level is sufficient to perform real-time data reconstruction, as the proposed recording frame rate is below 2 fps. In contrast, reconstructing the same image using the basis pursuit denoising algorithm (BPDN, a classic iterative CS algorithm)^26^ requires 3307.3 s on the same computer platform, nearly 7000 times slower than that of the proposed DNN. Doubling the image size to 1280 × 480 pixels further expands the diversity to 11,000 times (0.65 s for DNN and 7219.2 s for gradient projection), which is consistent with our prediction (see Supplementary Information S2 for details).

Second, a well-trained DNN has better denoising abilities, which can be readily obtained by adding more samples with noise at different levels during training. Moreover, adding noise has a regularization effect and, in turn, further improves the robustness of the model. In CS algorithms, however, denoising is largely experience-dependent. In general, a variable parameter is manually set during the iteration to cancel out the noise-induced bias. This operation works to some extent; however, it does not provide convincing results as long as the noise level changes. On average, the DNN enhances noise tolerance by 8.14 times under different noise levels (see Supplementary Information S3 for details).

### BEST camera in passive mode

We conducted a passive detection experiment to discern the target spectrum. Before illuminating the monochrome camera, the incident light from the target was modulated using random spectral filters (“Methods” and Supplementary Information S4). To quantify the spectral resolution of the proposed device, we measured the wavelength of a laser beam selected by an acousto-optic tunable filter (AOTF) from a white-light laser source. The bandwidth of the single-wavelength laser beam after AOTF was ~3.2 nm (full width at half maximum). Figure 1c–e presents a comparison of the reconstructed results from the proposed device (solid line) and the ground truth curves from a commercial spectrometer (Ocean Optics FLAME-S, dashed line). Two DNNs with identical frameworks were trained separately using two datasets (see Supplementary Information S5 for details), to guarantee the objectivity of the measurements. The first dataset contained only the spectrum information from the monochromatic light source, i.e., a laser beam. Moreover, the output spectrum from the trained DNN was strictly defined by a narrowband envelope. In this way (the “precise” mode), the average localization precision of the center wavelength in the reconstructed spectrum is 0.55 nm (Fig. 1c). In the “general” mode, we expanded the training dataset to include more broadband spectral data. In addition, we removed all the boundary conditions to support arbitrary spectrum shapes. This strategy shows comparable spectral localization precision at an average of 0.63 nm (Fig. 1d). However, it provides our camera with the generality to satisfy different operational needs. Such trained DNN allows a spectral resolution of ~5.2 nm (Fig. 1e), sufficient for most hyperspectral imaging applications in practice. Regarding this fact, we used the DNN working in “general” mode in the following experiments.

**Fig. 2:**
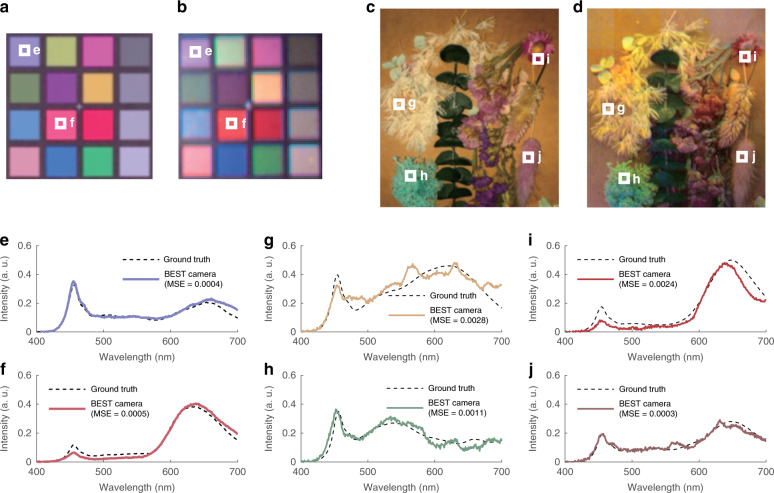
Applications of BEST camera in passive mode. **a** Photo of the color patches from the standard color calibration board. An RGB camera was used to take the photo. **b** Reconstructed spectral image (visualized in RGB form) of the color calibration card. Photo (**c**) and reconstructed spectral image (**d**) of practical plant sample. **e**–**j** Spectral profiles at the positions denoted by the white squares in **a**–**d**. The ground truths (measured by a commercial spectroradiometer, Konica Minolta CS-2000) and the DNN reconstructed results are represented by solid (reconstructed) and dashed (ground truth) curves, respectively

First, we used BEST camera to acquire a hyperspectral image from a standard color calibration card (X-Rite ColorChecker Classic Mini, Fig. 2a, b, e, f). For different colors, the mean square error (MSE) between the measured (solid) and ground truth (dashed) spectra ranged from 5 × 10^−6^ to 0.0032, with an average value of 0.0008. All spectra considered 16 color patches, as shown in Fig. 2a (see Supplementary Information S8). Compared to these artificially standard samples, natural objects, such as plants, represents a challenge for hyperspectral cameras. These samples usually contain rich structure and color details, requiring high spatial and high spectral resolutions to recover. However, the proposed hyperspectral camera overcomes this challenge by introducing DNN. Figure 2c, d, g–j show the results. The average reconstruction MSE is 0.0016, which is in good agreement with the previous experiment. However, the results are now obtained from a much more complex sample.

### BEST camera in active mode

**Fig. 3:**
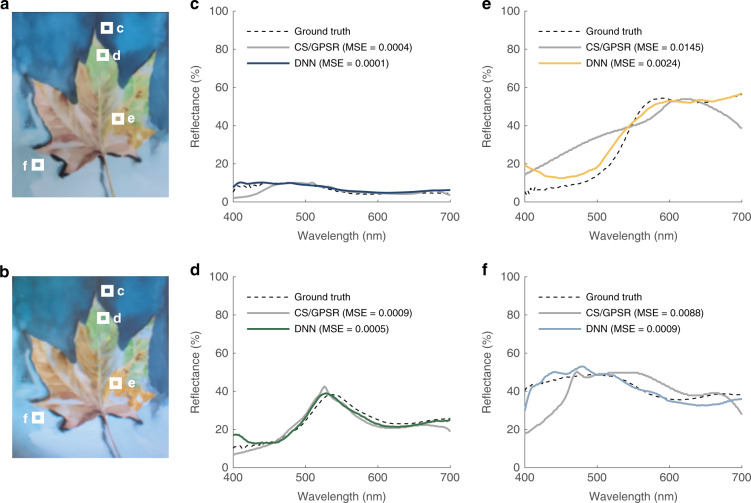
Applications of BEST camera in active mode. RGB photo (**a**) and reconstructed spectral images (**b**) of the watercolor painting sample. **c**–**f** Spectral profiles at the positions denoted by the white squares in **a**, **b**. The ground truths (measured by a commercial spectrophotometer, Olympus USPM-RU) and the DNN reconstructed results are represented by solid (reconstructed) and dashed (ground truth) curves, respectively

In addition to the passive model described above, the DNN can be applied to active hyperspectral imaging modes. Compared with passive cameras, active BEST cameras excel at measuring spectral reflectance regardless of the ambient illumination. To show the compatibility of the proposed DNN, we set up an active hyperspectral camera by placing a random spectral filter array in front of white-light light-emitting diodes (LEDs) to modulate the illumination light (“Methods” and Supplementary Information S6). We sequentially turned on each LED during the experiment and collected the reflected light from the target using a monochrome camera. We selected a watercolor painting (Fig. 3a) as the testing target. By feeding all captured images (a total of 16 frames) into the pre-trained DNN, we acquired its hyperspectral image, a 640 × 480 × 301 3D matrix. The results cover the entire visible range (400–700 nm) at a step size of 1 nm. We converted the spectral information into color space (Fig. 3b and “Methods”) to improve visualization. The retrieved image was highly consistent with the original target. The example spectral profiles from the labeled targets in Fig. 3b are presented in Fig. 3c–f. The MSE between the measured (solid) and ground truth (dashed) spectra was quantified at an average of 0.0010. Moreover, in good agreement with the simulations, the spectra reconstructed by the CS algorithm (gray lines in Fig. 3c–f) have an average MSE of 0.0061, 6.1 times worse than the DNN results.

The dimension of the active BEST cameras represents the major limitation. As the light source requires extra space, it is challenging to design and fabricate a compact active device. Nevertheless, benefitting from the advances of electronics technology, in particular, the surface-mounted devices, we compressed the dimensions of the proposed active BEST camera to 28.5 × 13.6 × 7.15 mm^3^ by miniaturizing the modulated illumination array and switching the monochrome charge-coupled device (CCD) camera to a cell-phone RGB complementary metal-oxide-semiconductor sensor (see Supplementary Information S7 for the details). These devices can potentially be integrated into wearables or cell phones for portable applications, such as electronics and health care.

## Discussion

The reliability of the output of the DNN was mainly determined by the spectral response of each random spectral filter. Any chromatic dispersion can severely worsen the reconstruction precision. The proposed random spectral filters were deposited by optical thin-film coating. A metasurface can also be considered to reduce the overlay steps during the production and pursue higher integration with the spectral camera. However, maintaining the optical properties such as transmittance/reflection spectra of the metasurfaces is challenging, especially when the polarization or the incident angle of light changes. The sensitivities to the polarization and incident angle, if not properly addressed, would narrow the application scale of hyperspectral cameras. Although the polarization effects are negligible for the thin-film random spectral filters used in the proposed system, the angular dispersion is still a force that should be considered. To compensate for the angular dispersion, we calibrated the spectral transmittance of each random spectral filter as a function of the light incident angle. In addition, we substituted them into the spectrum retrieval to improve the reconstruction precision (see Supplementary Information S9 for details). This strategy is beneficial for expanding the field-of-view or off-axis imaging, where most of the light with a large incident angle is present.

In summary, DNN enables us to capture hyperspectral images with a high spatial resolution (>10^5^ pixels) for complex samples and to develop an improved algorithm for spectral detection techniques. DNN allows a dramatically accelerated reconstruction to perform real-time spectral reconstruction and noise reduction. Compared with previous ones using CS algorithms, BEST camera excels in several aspects (see Supplementary Information S10 for details). Using our DNN to decode the spectrum from random spectral filters is only one of the several mechanisms in the computational spectroscopy family. In general, the algorithm presented in this study can be applied to techniques with similar purposes. This underscores the potential of applying our method to other computational spectroscopy techniques, such as linear X-ray spectroscopy and photoelectron spectroscopy^27^. This study expands the application of DNNs for optics. However, the functions discussed here, such as filter design, calculation acceleration, and de-noise, are just several possibilities. The entire potential of the technique is still to be realized.

Finally, the proposed system detects the spectrum at nanoscale resolution by computational spectroscopy and represents an important step toward artificial intelligence for optics.

## Methods

### DNN framework

The architecture of the DNN for spectral reconstruction can be described as “16-(LR-FC-500)^5^-LR-FC-301-LR.” Each number represents the number of units in the corresponding layer. LR indicates leaky ReLU units. FC denotes a fully connected layer, and the superscript “5” represents five repeat layers in the bracket. The input unit number corresponds to 16 random spectral filters and the output unit number 301 indicates the reconstructed spectral channels (400–700 nm, 1 nm step).

### Setup of passive BEST camera

In the passive BEST camera, the sample was imaged at random spectral filters through the relay lens. The encoded image was captured by a monochrome CCD camera (Vieworks VH-310G2) immediately behind the filter array. During the data acquisition process, 16 encoded images of the samples were captured. Each image corresponded to a specific filter of a random spectral filter array. Finally, the spectral data cube was reconstructed using DNN. A photograph of the experimental setup is shown in Supplementary Fig. S3.

### Setup of active BEST camera

In contrast to the passive one, the random spectral filters in the active BEST camera were placed in front of the white-light LEDs (Luminus MP-3030-1100-56-95) to tune the illumination light. During the data acquisition process, each LED was sequentially turned on. A monochrome CCD camera (Vieworks VH-310G2) was used to collect the reflected light from the target. As the encoding manner changes, the DNN is retrained using the data collected by the active mode. A photograph of the experimental setup is shown in Supplementary Fig. S5.

### PCSED and filter design

During the filter design, PCSED focused on solving the following problem:

$$(\hat P,\hat \theta ) = \mathop {{{\mathrm{argmin}}}}\limits_{P,\theta } ||{\mathbf{s}} - {\cal{D}}_\theta [{\cal{F}}{\cal{M}}\left( P \right)^T \cdot {\mathbf{s}}]||_2^2 + R(P)$$

where **s** represents the spectrum in discrete form, ${\cal{F}}{\cal{M}}$ denotes the forward modeling network (FMN), and *P* is the filter structure parameter. ${\cal{D}}_\theta$ represents the DNN for spectral reconstruction with network parameters *θ* and *R*(*P*) is the regularization term constraining the structural parameters, which, in this case, includes the thin-film thickness and coating angles. The initial parameters of *P* comprise 16 angles and 30 thicknesses. The FMN maps the parameters *P* to the corresponding spectral responses theoretically calculated from 30 layers of SiO_2_ and TiO_2_ at 16 coating angles. By solving the PCSED problem with a dataset of 1,000,000 spectra from Computer Vision Laboratory (CAVE) and Interdisciplinary Computational Vision Laboratory (ICVL), a set of structural parameters directly used by the coating machine was obtained. Moreover, the DNN for spectral reconstruction was also trained. All 16 filters were coated for faster fabrication in a single deposition process using an electronic beam evaporator (Optorun OTFC-1300). More details on the filter fabrication are available in Supplementary Information S11.

### Visualization of hyperspectral image

The direct visualization of the hyperspectral image was allowed by converting the spectra into CIEXYZ color space by calculating the *X*, *Y*, and *Z* tristimulus values according to:

$$\left\{ {\begin{array}{*{20}{c}} {X = \mathop {\smallint }\limits_{\lambda 1}^{\lambda 2} S\left( \lambda \right)\bar x\left( \lambda \right)d\lambda } \\ {Y = \mathop {\smallint }\limits_{\lambda 1}^{\lambda 2} S\left( \lambda \right)\bar y\left( \lambda \right)d\lambda } \\ {Z = \mathop {\smallint }\limits_{\lambda 1}^{\lambda 2} S\left( \lambda \right)\bar z\left( \lambda \right)d\lambda } \end{array}} \right.$$

where $\lambda _1$ and $\lambda _2$ represent the wavelength range, $S\left( \lambda \right)$ is the spectrum of each pixel, and $\bar x$, $\bar y$, and $\bar z$ are the standard observer color-matching functions. The calculated (*X*, *Y*, *Z*) was further converted into an sRGB color space to display using the MATLAB function “xyz2rgb.”

## Supplementary information

Supplementary Information

## References

[1] Gabrieli F (2019). Near-UV to mid-IR reflectance imaging spectroscopy of paintings on the macroscale. Sci. Adv..

[2] Bahauddin SM, Bradshaw SJ, Winebarger AR (2021). The origin of reconnection-mediated transient brightenings in the solar transition region. Nat. Astron..

[3] Williams LJ (2021). Remote spectral detection of biodiversity effects on forest biomass. Nat. Ecol. Evol..

[4] Zhang DL (2016). Depth-resolved mid-infrared photothermal imaging of living cells and organisms with submicrometer spatial resolution. Sci. Adv..

[5] Greaves, J. S. et al. Phosphine gas in the cloud decks of Venus. *Nature Astronomy*, 10.1038/s41550-020-1174-4 (2020).

[6] Pavia, D. L. et al. *Introduction to Spectroscopy* (Cengage Learning, 2014).

[7] Hagen NA, Kudenov MW (2013). Review of snapshot spectral imaging technologies. Opt. Eng..

[8] Cao X (2016). Computational snapshot multispectral cameras: toward dynamic capture of the spectral world. IEEE Signal Process. Mag..

[9] Gehm ME (2007). Single-shot compressive spectral imaging with a dual-disperser architecture. Opt. Express.

[10] Cai, X. S. et al. One-shot ultraspectral imaging with reconfigurable metasurfaces. Preprint at https://arxiv.org/abs/2005.02689 (2020).

[11] Wang Z (2019). Single-shot on-chip spectral sensors based on photonic crystal slabs. Nat. Commun..

[12] Oliver J, Lee WB, Lee HN (2013). Filters with random transmittance for improving resolution in filter-array-based spectrometers. Opt. Express.

[13] Bao J, Bawendi MG (2015). A colloidal quantum dot spectrometer. Nature.

[14] Yang ZY (2019). Single-nanowire spectrometers. Science.

[15] Baraniuk RG (2007). Compressive sensing. IEEE Signal Process. Mag..

[16] Choi I (2017). High-quality hyperspectral reconstruction using a spectral prior. ACM Trans. Graph..

[17] Wang, L. et al. Hyperspectral image reconstruction using a deep spatial-spectral prior. In *Proc. IEEE/CVF Conference on Computer Vision and Pattern Recognition* 15–20 June 2019 (IEEE, Long Beach, CA, USA, 2019).

[18] Gewali UB, Monteiro ST, Saber E (2019). Spectral super-resolution with optimized bands. Remote Sens..

[19] Nie, S. J. et al. Deeply learned filter response functions for hyperspectral reconstruction. In *Proc. IEEE/CVF Conference on Computer Vision and Pattern Recognition* 18–23 June 2018 (IEEE, Salt Lake City, UT, USA, 2018).

[20] So S (2020). Deep learning enabled inverse design in nanophotonics. Nanophotonics.

[21] Voznyy O (2019). Machine learning accelerates discovery of optimal colloidal quantum dot synthesis. ACS Nano.

[22] Gao L (2019). A bidirectional deep neural network for accurate silicon color design. Adv. Mater..

[23] Liu DJ (2018). Training deep neural networks for the inverse design of nanophotonic structures. ACS Photonics.

[24] Song HY (2021). Deep-learned broadband encoding stochastic filters for computational spectroscopic instruments. Adv. Theory Simul..

[25] Goodfellow I, Bengio Y, Courville A (2016). Deep Learning.

[26] Figueiredo MAT, Nowak RD, Wright SJ (2007). Gradient projection for sparse reconstruction: application to compressed sensing and other inverse problems. IEEE J. Sel. Top. Signal Process..

[27] Alford, T. L., Feldman, L. C. & Mayer, J. W. *Fundamentals of Nanoscale Film. Analysis* (Springer, 2007).

