# Supplementary material for: Deeply learned broadband encoding stochastic hyperspectral imaging
Source: Light Sci Appl. 2021 May 25;10:108. doi: 10.1038/s41377-021-00545-2 (PMC8149860; doi:10.1038/s41377-021-00545-2)
Supplement: Supplementary file 1 — Supplementary Information [file 41377_2021_545_MOESM1_ESM.docx]

**Supplementary Information for**

**Deeply Learned Broadband Encoding Stochastic Hyperspectral Imaging**

**Authors**

Wenyi Zhang^1#^, Hongya Song^1#^, Xin He^1^, Longqian Huang^1^, Xiyue Zhang^1^, Junyan Zheng^1^, Weidong Shen^1^, Xiang Hao^1*^, and Xu Liu^1*^

**Affiliations**

^1^ State Key Laboratory of Modern Optical Instrumentation, College of Optical Science and Technology, Zhejiang University, Hangzhou 310027, China

^#^ These authors contribute equally to this work.

^*^ Correspondence and requests for materials should be addressed to X. Hao ([haox@zju.edu.cn](mailto:haox@zju.edu.cn)) and X. Liu ([liuxu@zju.edu.cn](mailto:liuxu@zju.edu.cn))

**S1. Spectra of random spectral filters**


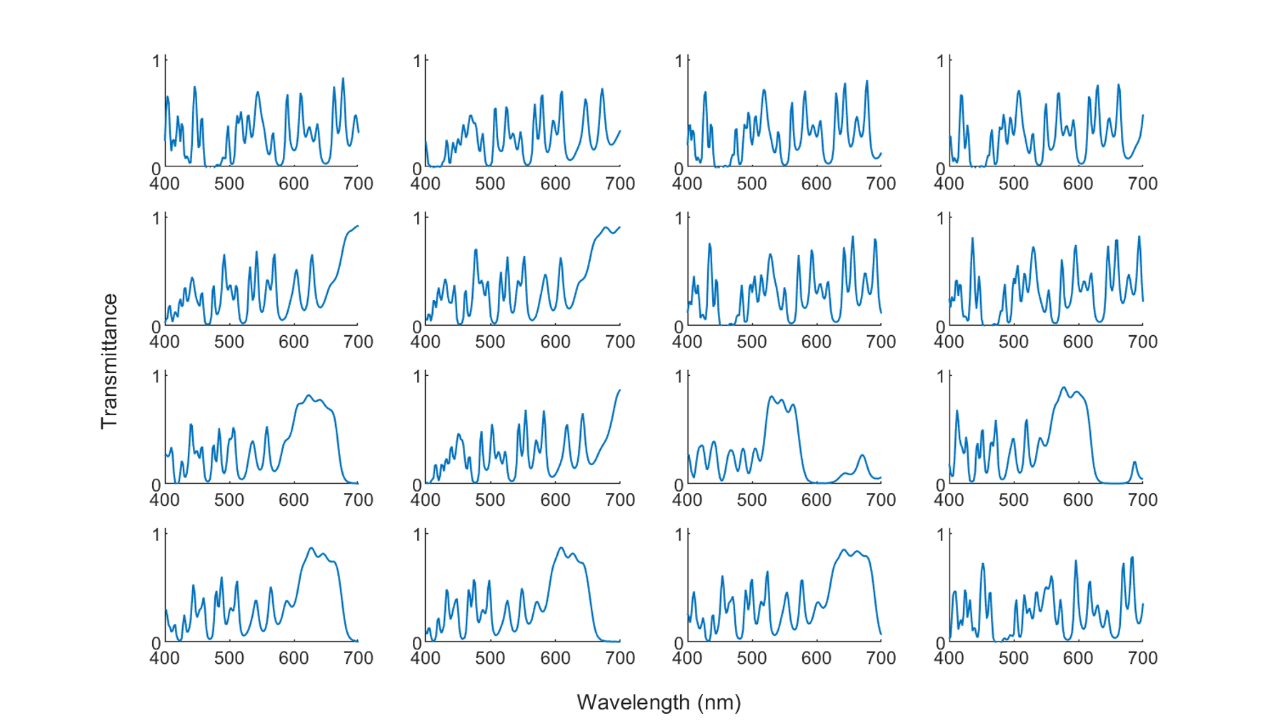


**Fig. S1 Spectral transmittance of fabricated random spectral filters.**

**S2. Reconstruction speed**

To compare the speed of DNN and iterative CS algorithm, we reconstructed the spectral images of different sizes on an Intel Core i9-10900x CPU, Nvidia GeForce 2080Ti GPU platform. The measured time consumption is shown in Table. S1. In general, the speed of DNN is almost irrelative to the spatial resolution, while the consumption time of the CS algorithm and the pixel number roughly follow a linear relationship. Running iterative CS algorithm is inefficient on GPU because data exchanging is frequently needed for iteration, but a single GPU core is much slower than a CPU core. The memory of the GPU limits the maximum number of parallel computing pixels. In our case, the upper limit of the pixel number is around 650,000 for 11 GB of memory.

**Table S1. Reconstruction time of ultra-spectral image at different spatial resolutions**

|  | CS/s | DNN/s |
| --- | --- | --- |
| 480×320 | 1643.5 | 0.41 |
| 480×640 | 3307.3 | 0.48 |
| 480×1280 | 7219.2 | 0.65 |

The reconstruction speed can be further improved by employing an advanced GPU or running multiple GPUs simultaneously. Notably, most memory is used to store the output spectra. Therefore, to avoid the tradeoff between spectral and spatial resolutions, memory capability is as important as the number of GPU cores.

**S3. Performance under noises**

In the CS signal recovery, a classical reconstruction algorithm is the gradient projection for sparse reconstruction ^1^. In brief, it solves the convex optimization problem of basis pursuit de-noising (BPDN):

$$\min_{\mathbf{x}} \frac{1}{2}\left\| \mathbf{y}-A\mathbf{x} \right\|_{2}^{2}+\tau\left\| \mathbf{x} \right\|_{1}$$

where **x** is the sparse form of the original signal, *A* is the product of the sensing matrix and sparse basis, and **y** is the measured signal. By solving the optimization problem the original signal can be reconstructed by the production of sparse vector **x** and sparse basis. The non-negative parameter *τ* also influences the reconstruction accuracy under noisy conditions, so it must be carefully selected. Meanwhile, to maximize the reconstruction precision, which can be quantified with mean square error (MSE) or peak signal-to-noise ratio (PSNR), *τ* has to be given diverse values (Fig. S2A). Since the noise level prediction before the experiment is impossible in practice, choosing a proper *τ* becomes challenging and highly relies on the experience, which reduces the generality of the CS algorithm.

We compared the performance of DNN and the BPDN. Under the noise levels from 1% to 5%, without any human interoperation, the DNN firmly provides higher PSNR even compared to the CS/BPDN algorithm with the optimal *τ*. On average, the PSNR of DNN is 9.1 dB higher, equivalent to 8.14 folds of MSE (Fig. S2B).


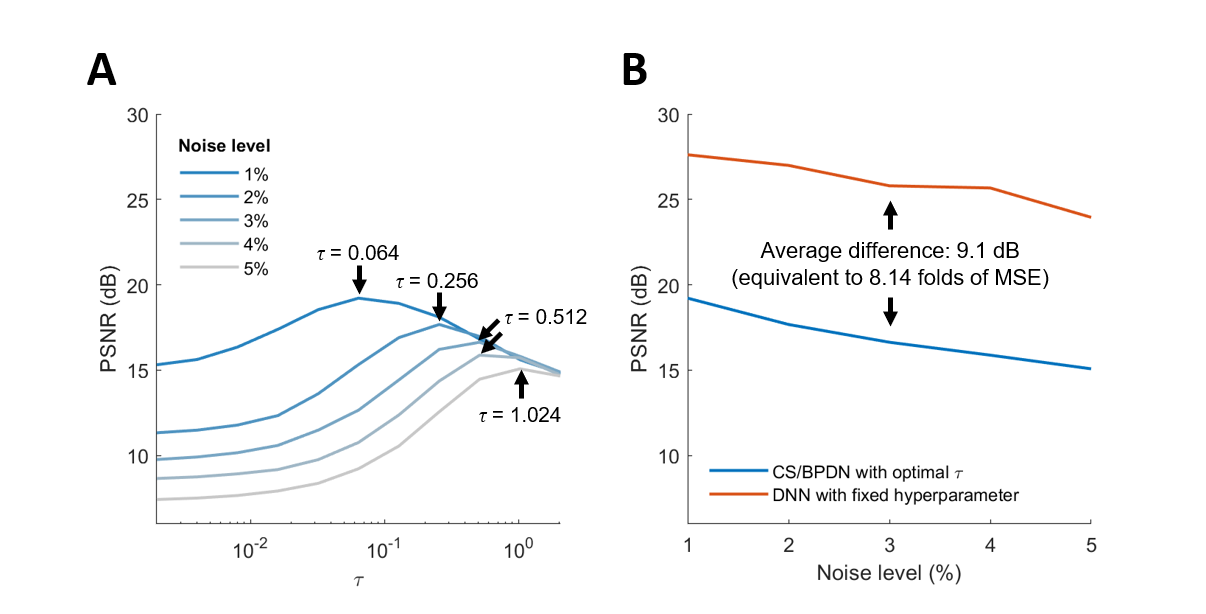


**Fig. S2. De-noise effects of different algorithms. (A)** Average PSNR for BPDN (CS algorithm) as a function of τ. **(B)** Average PSNR for DNN and BPDN algorithms for signals at different noise levels.

**S4. Setup of passive BEST camera**

The passive modality of our BEST camera is shown in Fig. S3.


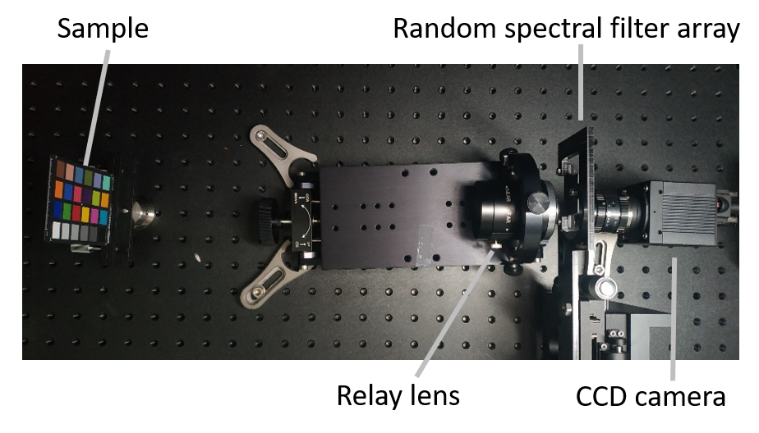


**Fig. S3. Experimental setup of passive BEST camera.**

**S5. Training details of the DNN for spectral reconstruction**

The “precise” dataset for training DNN was constructed with 200,000 narrowband spectra, of which the center wavelength of the spectra are randomly generated. On the other hand, the “general” dataset is an extension of the “precise” dataset, which also includes the broadband spectra of the pixels taken from the spectral images of the CAVE ^2^ and the ICVL ^3^ datasets. The ratio of the narrowband and broadband spectra in the dataset is 1:1, and the total number reaches 400,000. The input of the DNN is the normalized intensity signal obtained behind 16 random spectral filters, and the luminance information is maintained by recoding the normalizing factor. Both DNNs were trained on an Nvidia GeForce RTX2080Ti GPU platform. Training “precise” and “general” DNN cost 6’3” (363 seconds) and 11’48” (708 seconds), respectively. The training and test loss vs. epoch curve is shown in Fig. S4.

The measured spectra of DNN working in “precise” mode are assumed to have a narrowband shape. Adding this prior dramatically reduces the volume of output data from several hundred to two values (one center wavelength value and one FWHM value). Therefore, it is beneficial for improving the convergence, and in turn, increasing the spectral resolution. The similar strategy was used in other researches ^4,5^. However, any reconstruction algorithm based on this assumption is hardly applicable to the broadband spectra. For most spectral imaging purposes, the DNN trained by the “general” dataset is still an essence. Taking this fact into account, we believe the spectral resolution claimed by the “general” DNN can present the spectral reconstruction more faithfully.


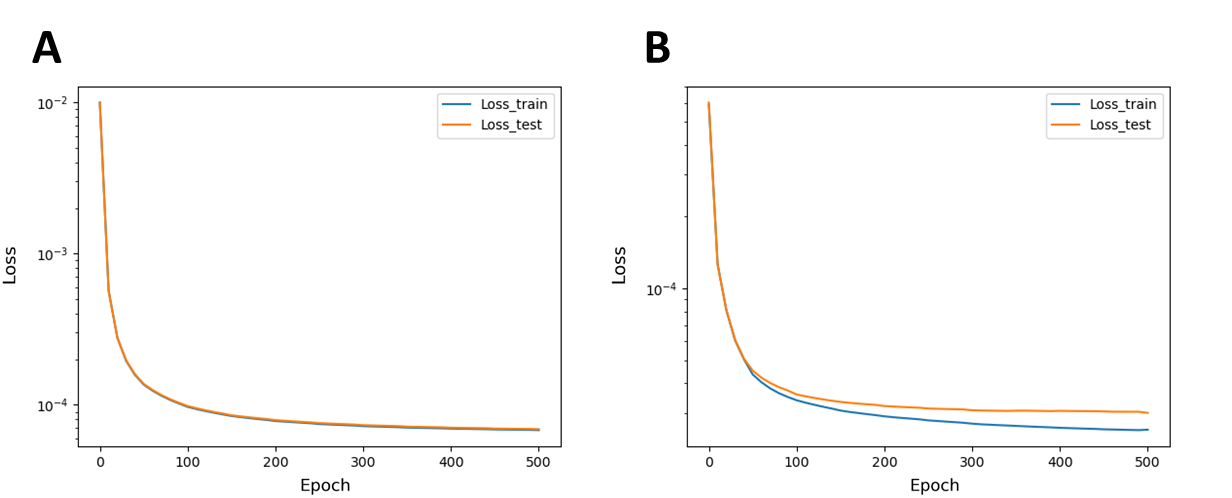


**Fig. S4. Training and test loss vs. epoch curve.** **(A)** “precise” mode, **(B)** “general” mode.

**S6. Setup of active BEST camera**

The active modality of our BEST camera is shown in Fig. S5.


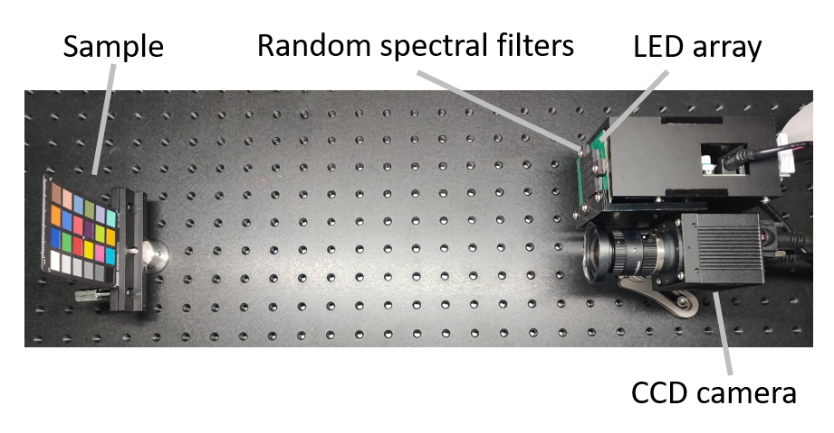


**Fig. S5. Experimental setup of active BEST camera.**

**S7. Compact active BEST camera design**

To compress the dimensions of our active system, we miniaturize the modulated illumination array and switch the monochrome CCD camera to a cell-phone RGB complementary metal-oxide-semiconductor (CMOS) sensor (SONY IMX380). The random spectral filters are cut into 1.6×1.6 mm^2^ pieces and stuck to a holder fabricated with wire electrical discharge machining. The holder is opaque and separates the filters to minimize the crosstalk when the LEDs (Everlight ELCS14B-NB5060J7J9283910-F4Z) are sequentially lightened. Compared with those in the original active BEST camera, these LEDs are smaller in size (1.38×1.38 mm^2^). The LEDs and the CMOS camera were welded on a microcircuit board, and the filter array was integrated onto the LED array. The microcircuit was powered and controlled through a flexible flat cable by an android-based RK3399 Pro demo board. The program running on this board synchronizes the lighting of the LEDs and the capturing of the CMOS camera, allowing the reconstruction of the spectra directly using the captured data. The photo of our compact active BEST camera is shown in Fig. S6.

The camera has a color image sensor with three kinds of pixels: red (R), green (G), and blue (B), representing three different spectral responses. These color filters are arranged in an RGGB Bayer filter array. To adapt the DNN with the compact design, we interpolated the raw images to directly read all RGB intensities. For every pixel, we summed up three intensities as the overall intensity, corresponding to the intensity captured by a monochrome charged coupled device (CCD). Three spectral responses were also added as one curve to replace the quantum absorption efficiency of CCD. In this way, the DNN training process can be directly used for our compact design by generating another training dataset using CMOS spectral responses.


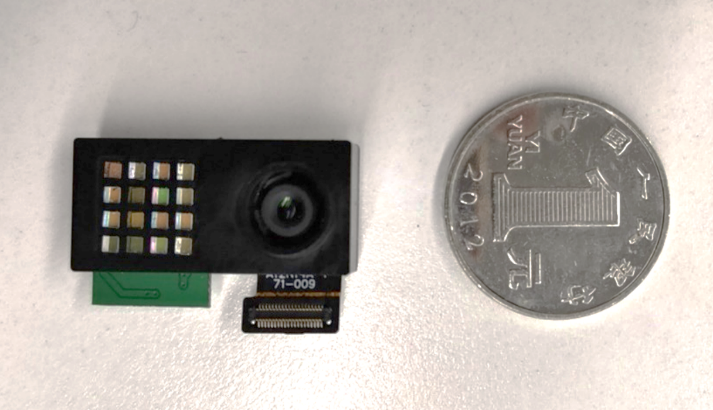


**Fig. S6. Photo of the compact active BEST camera.**

**S8. Spectra of all color patches in Fig. 2A**


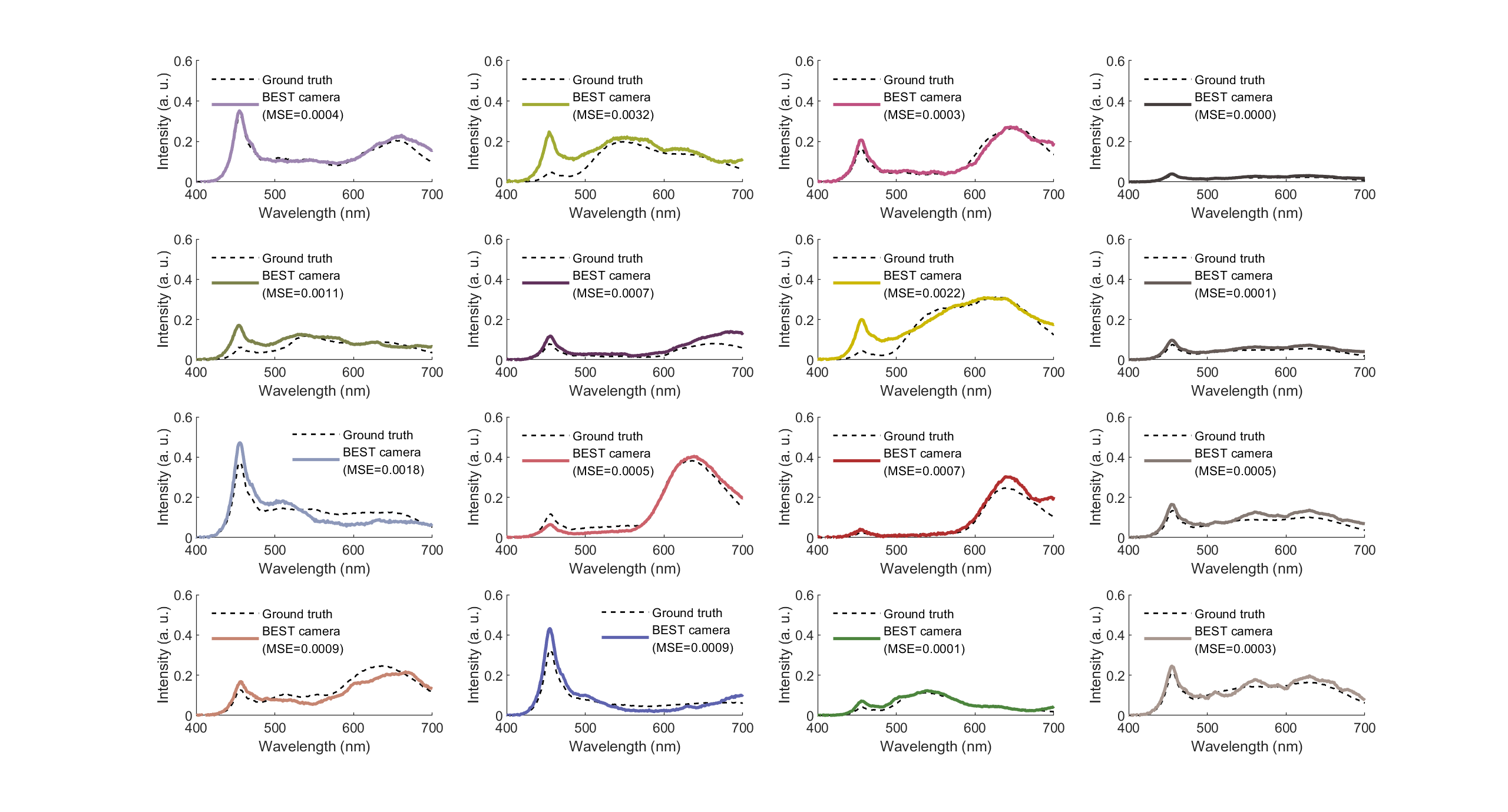


**Fig. S7. Ground truth and measured spectra of all color patches in Fig. 2A.** Each plot represents the spectrum of the patch at the corresponding position in main text Fig. 2A. All patches are illuminated by a 5600K LED light source.

**S9. Compensation for angular dispersion**

The light rays from different field of view (FOV) but collected by the same pixel have different incident angles when they propagate the random spectral filters. As these filters are angle-sensitive in transmission mode, the angular chromatic dispersion deviates the transmittance from those used in the training process, inducing reconstruction errors. To compensate for the angular dispersion, we first calibrated the spectral transmittance of each filter as a function of incident angle. Then we trained the DNN using these variable functions, resulting in a group of angle-dependent DNNs. During the spectrum retrieval, for each pixel, the exact incident angle was pre-calculated by analyzing the relative positions of the light source, the sample, and the camera. This angle information further enabled us to enhance the robustness of our algorithm.

To demonstrate our ability to compensate for the angular dispersion, we placed a single-color sample at different positions with different angles. The spectrum of this sample was then calculated using the DNN with and without angular compensation. The comparison of reconstruction results using and without using angular compensation is shown in Fig. S8. The reconstructed spectra without angular compensation have an approximate 10 nm peak wavelength shift (Fig. S8A). This shift was completely gone after we introduced the compensation strategy (Fig. S8B).


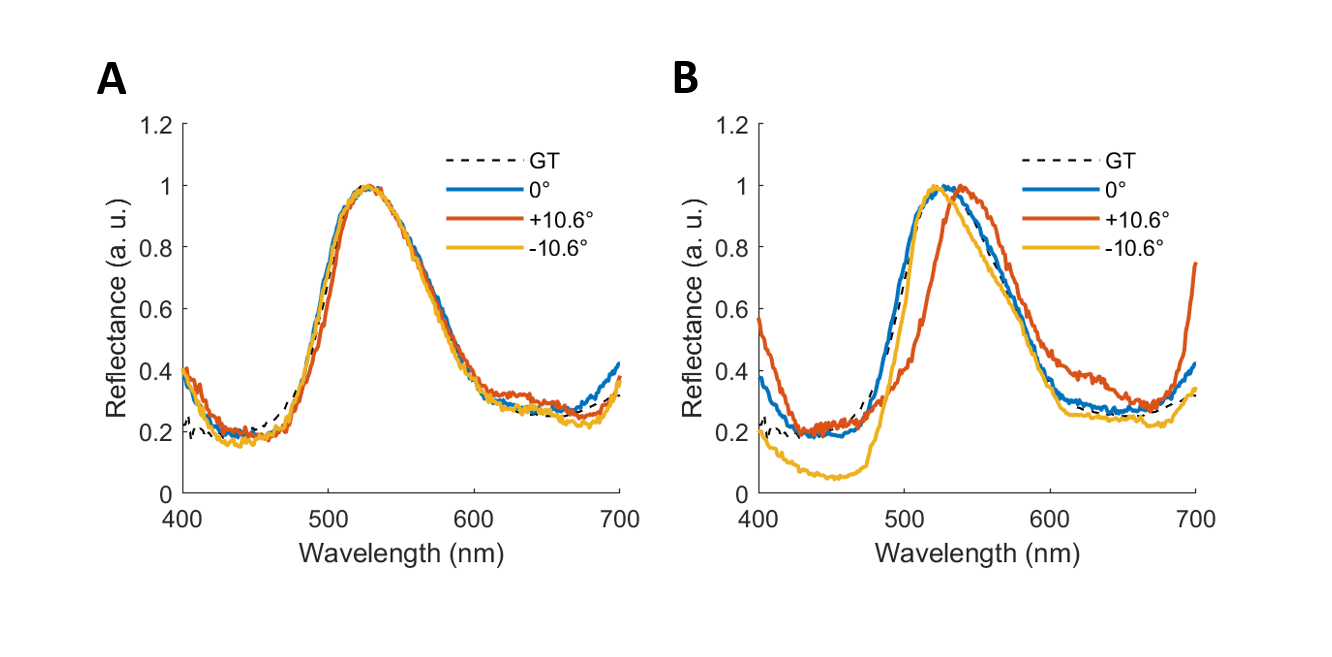


**Fig. S8. Comparison between reconstruction results.** **(A)** with angular compensation, **(B)** without angular compensation.

**S10. Performance comparison with previous reports**

**Table S2. Performance comparison with previous reports on critical parameters**

|  | Spectral resolution | Spectral range | Pixel number | Output data volume | Reconstruction time |
| --- | --- | --- | --- | --- | --- |
| Ref. ^5^ | Not available | 550–750 nm | 10*10 | ~10^4^ | Not available |
| Ref. ^6^ | 10–15 nm | 500–630 nm | 20*10 | ~10^4^ | Not available |
| Ours (CS/BPDN) | 10 nm | 400–700 nm | 640*480 | ~10^8^ | 3307.3 s |
| Ours (DNN) | 5.2 nm | 400–700 nm | 640*480 | ~10^8^ | 0.48 s |
| Ref. ^7^ CASSI (sparse coding) | 10 nm | 420–720 nm | 512*384 | ~10^7^ | 30,636 s |
| Ref. ^8^ CASSI: (spectral prior) | 10 nm | 420–720 nm | 512*512 | ~10^7^ | 414 s |
| Ref. ^9^ CASSI (spatial-spectral prior) | 10 nm | 400–700 nm | 512*512 | ~10^7^ | 1.11 s |

Note that the spectral resolution of our system is analyzed by the nearest distinguishable center wavelength of double-peak spectra. The output data volume is evaluated by the size of the hyperspectral datacube, i. e. pixel number times the spectral channel. For example, the output data volume of our system is 640*480*301, which is equivalent to ~10^8^.

**S11. Filter fabrication details**

For each filter, there are 30 thin-film SiO_2_ and TiO_2_ layers alternately coated. The thicknesses of 30 layers in each filter determines the corresponding spectral response. To realize different spectral responses, all the 16 filters must have unique thin-film structure, *i. e.* a unique group of the 30 thickness values. In conventional coating process, the thickness of each thin-film layer is controlled by the evaporating time of the dielectric material (SiO_2_ and TiO_2_), and one continuous coating process only generates one kind of filter. As a consequence, 16 different filters have to be made by 16 different coating process. To reduce the time consumption and the complexity of coating, we aim to fabricate all 16 filters in one coating process. Therefore, we place the 16 filters in 16 holders with different angles so that the thickness of thin-film layers differs not only by the evaporating time, but also the angles. Ideally, the coated thickness $t$ follows equation $t=t_{0}\cos\theta$, where $t_{0}$ is the thickness of a certain layer coated on the 0 degree holder. In this way, we can get 16 filters with different structures in one continuous coating process, and therefore, we consider the angle of 16 holders as well as the 30 thicknesses of the layers on the 0 degree holder as the design parameters.

**References**

1 Chen, S. S. B., Donoho, D. L. & Saunders, M. A. Atomic decomposition by basis pursuit. *Siam Journal on Scientific Computing* **20**, 33-61 (1998).

2 Yasuma, F. et al. Generalized Assorted Pixel Camera: Postcapture Control of Resolution, Dynamic Range, and Spectrum. *Ieee Transactions on Image Processing* **19**, 2241-2253 (2010).

3 Arad, B. & Ben-Shahar, O. in *European Conference on Computer Vision.* 19-34 (Springer, Cham, 2016).

4 Oliver, J., Lee, W. B. & Lee, H. N. Filters with random transmittance for improving resolution in filter-array-based spectrometers. *Optics Express* **21**, 3969-3989 (2013).

5 Wang, Z. *et al*. Single-shot on-chip spectral sensors based on photonic crystal slabs. *Nature Communications* **10**, 1020 (2019).

6 Yang, Z. Y. *et al*. Single-nanowire spectrometers. *Science* **365**, 1017-1020 (2019).

7 Lin, X. *et al*. Spatial-spectral encoded compressive hyperspectral imaging. *ACM Transactions on Graphics* **33**, 1-11 (2014).

8 Choi, I. *et al*. High-quality hyperspectral reconstruction using a spectral prior. *ACM Transactions on Graphics* **36**, 218 (2017).

9 Wang, L. *et al*. Hyperspectral image reconstruction using a deep spatial-spectral prior. Proceedings of the IEEE/CVF Conference on Computer Vision and Pattern Recognition; 15–20 June 2019; Long Beach, CA, USA. Long Beach, CA, USA: IEEE, 2019.
